# Junín Virus Pathogenesis and Virus Replication

**DOI:** 10.3390/v4102317

**Published:** 2012-10-22

**Authors:** Ashley Grant, Alexey Seregin, Cheng Huang, Olga Kolokoltsova, Allan Brasier, Clarence Peters, Slobodan Paessler

**Affiliations:** 1 Department of Pathology, University of Texas Medical Branch, Galveston, TX 77555, USA; Email: amgrant@utmb.edu (A.G.); alseregi@utmb.edu (A.S.); chhuang@utmb.edu (C.H.); oakoloko@utmb.edu (O.A.); cjpeters@utmb.edu (C.P.); slpaessl@utmb.edu (S.P.); 2 Institute for Translational Sciences, Department of Internal Medicine and Sealy Center for Molecular Medicine, University of Texas Medical Branch, Galveston, Texas; Email: arbrasie@utmb.edu

**Keywords:** arenavirus, Junín virus, pathogenesis

## Abstract

Junín virus, the etiological agent of Argentine hemorrhagic fever, causes significant morbidity and mortality. The virus is spread through the aerosolization of host rodent excreta and endemic to the humid pampas of Argentina. Recently, significant progress has been achieved with the development of new technologies (e.g. reverse genetics) that have expanded knowledge about the pathogenesis and viral replication of Junín virus. We will review the pathogenesis of Junín virus in various animal models and the role of innate and adaptive immunity during infection. We will highlight current research regarding the role of molecular biology of Junín virus in elucidating virus attenuation. We will also summarize current knowledge on Junín virus pathogenesis focusing on the recent development of vaccines and potential therapeutics.

## 1. Introduction

Arenaviral infections are frequent causes of acute disease in humans. Junín virus (JUNV) causes Argentine hemorrhagic fever (AHF), a disease endemic to the pampas region of Argentina, with approximately five million people at risk [1,2]. AHF was first described in 1953, and the virus was isolated several years later.

## 2. Agent

JUNV is an enveloped virus with a bi-segmented negative stranded RNA genome [3]. The genomic RNA segments, large (L) (7.3 kb) and small (S) (3.5 kb), use an ambisense coding strategy to encode two open reading frames in opposite orientation, separated by a non-coding intergenic region that acts as a transcription termination signal for the viral polymerase [4,5]. The S RNA segment encodes the viral glycoprotein precursor (GPC) and the nucleoprotein (NP). GPC is post-translationally cleaved by the cellular subtilase SKI-1/S1P to yield the two glycoproteins, GP-1 and GP-2, and the signal stable peptide (SSP). The glycoproteins are embedded in the lipid bilayer to form the viral spikes in the mature virion that are crucial for receptor recognition and virus entry while the SSP is responsible for modulating the response of GPC to acidic pH [6,7,8]. The L RNA segment encodes the viral RNA-dependent RNA polymerase (L polymerase) [9] and the small (11 kDa) RING finger protein Z that is the arenavirus equivalent to the matrix protein found in many other negative strand RNA viruses [10,11,12].

**Figure 1:**
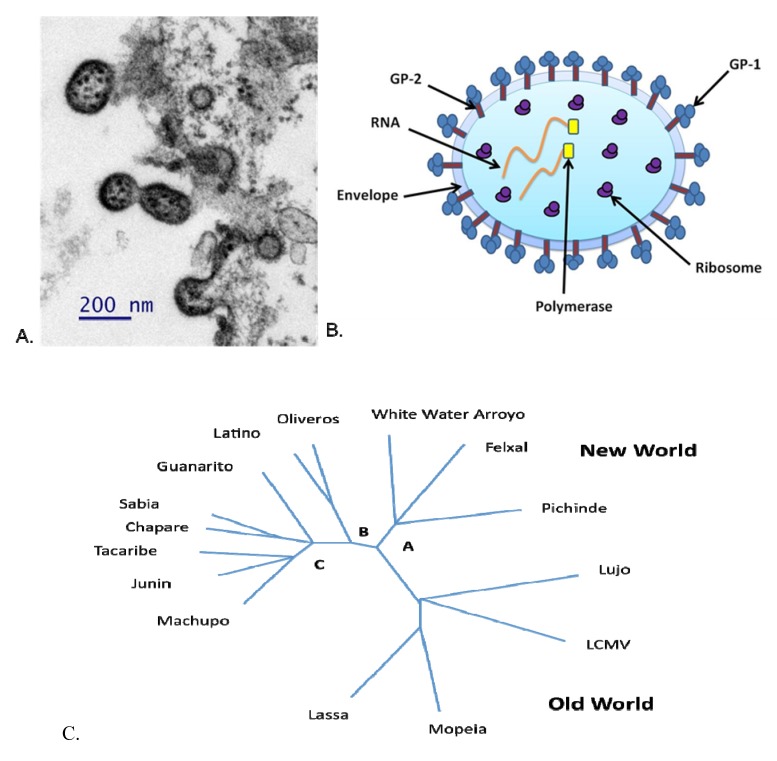
(a) Thin section transmission electron microscopy photomicrograph of Junín virus (JUNV) Candid#1 budding from the surface of Vero E6 cells. (b) Structure of a JUNV particle. (c) Phylogenetic tree of the *Arenaviridae* family. The top half of the figure shows the New World arenaviruses and the bottom half shows the Old World arenaviruses. The New World arenaviruses are separated into three different clades (A, B, and C). Genetic variation is depicted as distance. Adapted from [13].

## 3. Epidemiology

JUNV causes chronic infections of rodents, which are mobile in their natural habitat. Humans are infected through mucosal exposure, aerosols, or by direct contact of abraded skin with infectious material. Person-to-person transmission is very rare and may occur via direct contact with infected body fluids of a viremic patient, and nosocomial infections have been reported [3]. Major epidemics occur predominantly during the harvesting season in Argentina, with a peak incidence in the month of May. The disease is four times as prevalent in males as in females, and is more prevalent among rural workers than in urban populations. The annual incidence of AHF is positively correlated with local population densities of the reservoir, the drylands vesper mouse (*Calomys musculinus*) [1]. This epidemiologic pattern of disease is further modified through vaccination of high-risk populations.

## 4. Clinical Manifestations, Pathology and Pathogenesis

The onset of the clinical disease is similar to many infectious diseases, with malaise, anorexia, chills, headache, myalgia, and fever (38°C to 39°C). Patients may progress to develop constitutional, gastrointestinal, cardiovascular and neurologic signs and symptoms several days later. Symptoms reported include: malaise, retroorbital pain, nausea, vomiting, epigastric pain, photophobia, dizziness, petechiae, and constipation or mild diarrhea.

Reverse transcription–polymerase chain reaction (RT–PCR) is usually the most sensitive diagnostic assay and produces amplicons that can be sequenced for genetic analysis. In general, viremia and antigenemia are readily detected during the acute phase of the disease and disappear during convalescence. The presence of viral nucleic acid can be detected during the same period and sometimes several days longer [14]. Seroconversion, mainly IgM antibodies, may be detected during illness and usually appear early in convalescence.

During AHF, hemorrhages may or may not be present in different organs, and effusions can be found in serous cavities. In general, widespread necrosis is present in any organ and varies from modest and focal to massive and multifocal. Liver and lymphoid systems are usually extensively involved, and the lungs regularly have varying degrees of interstitial pneumonitis, diffuse alveolar damage, and hemorrhage. The typical inflammatory response found in AHF is usually minimal. Treatment with immune plasma infusion can sometimes lead to late neurologic syndrome (LNS) where patients experience cerebellar manifestations and a fever.

AHF during pregnancy is uncommon, but in the last trimester more than half of patients succumb to the disease, at least in part due to tardy recognition of the disease and failure to administer specific treatment. Congenital malformations and fetal and neonatal deaths also occur [14,15]. Children tend to have a milder clinical course, but severe and even fatal disease has been reported.

Inhalation of JUNV particles following aerosolization likely results in uptake by alveolar macrophages and is followed by migration of infected macrophages to draining lymph nodes. As the virus replicates, it disseminates through the vascular system to the kidney, adrenal, lungs, vascular endothelium, central nervous system, and lymphoid organs. 

Macrophages are critical cellular targets in arenavirus infection [16]. In a published case series, macrophages from seven fatal cases of AHF showed cytopathic effects, virions were observed budding from the plasma membrane, and viral antigen was demonstrated by immunofluorescence [17]. Splenic white pulp and lymph node destruction was also observed in fatal AHF cases, clearly illustrating viral tropism for lymphatic tissue [17]. Macrophage tropism of JUNV could be an important mechanism of immune system evasion and play a role in the fatal shock syndrome that occurs in some patients with AHF. Previous studies have shown that virulent Pichinde (PCV) and Lassa (LASV) infection do not result in activation of macrophages; however, the cells are still a main target of the infection [18,19]. Another study with an Old World arenavirus showed survival following LASV infection is correlated with the presence of inflammatory cytokines in the plasma [20]. The same may be true for JUNV infection. LASV and JUNV are non-cytopathic *in vitro* compared to a lytic infection such as the one observed with Rift Valley fever virus [21]. LASV and JUNV can replicate in primary endothelial cells as well as human macrophages and monocytes without overt cellular damage [22]. Likewise, infection of Schwann cells with lymphocytic choriomeningitis virus (LCMV) and LASV failed to result in apoptosis or cytopathic effects [23]. The major tropism of pathogenic arenaviruses is macrophages yet it appears the infection is not lytic unlike other VHFs. 

New World arenavirus clearance appears to be primarily mediated via a humoral immune response [24]. Due to the minimal CPE in different tissue culture monolayers, inflammatory mediators from macrophages are thought to be the effectors of the disease outcomes in New World arenaviruses [25]. The majority of hemorrhaging during AHF appears to be due to inhibition of platelet aggregation and thrombocytopenia.

## 5. Animal Models

### 5.1 JUNV Infection in Mice

Recent studies have showed that mice that have deficiencies in the innate immune system can be infected with JUNV [26]. New born mice intracranially (i.c.) infected with JUNV develop fatal encephalitis, which is prevented in athymic, neonatally thymectomized or immunosuppressive drug /serum administrated mice [27,28]. Accordingly, an immunopathological mechanism is thought to be involved in the pathogenicity of JUNV infection in the mouse model [29].

Adult mice infected i.c. with JUNV have high virus titers in the brain for over two weeks but do not show symptomatic disease. Adult C3H/HeJ mice, a mouse strain lacking toll like receptor (TLR)-4, are susceptible to Junín XJ strain when infected i.c., causing a high mortality rate within 13 days with no neutralizing antibodies and apparent delayed-type hypersensitivity reaction. This strain is not susceptible to other routes of infection outside the central nervous system (CNS) [30]. TLR-4 is responsible for pathogen recognition and activation of the innate immune system. TLR-4 knockout mice susceptibility shows the importance of the innate immune system activation in Junín virus mouse model infection. Newborn mice experience a lethal meningoencephalitis induced by delayed type hypersensitivity (DTH) and adult mice are susceptible when treated with cyclophosphamide resulting in immunosuppresion [31]. Newborn athymic nude mice with i.c. injection of 1000 TCID_50_ of Junín XJ 24 to 48 hours after birth survive, while control mice show a mortality rate of 92%. Both sets of mice have 7–8 logs of virus in the brain at seven days p.i.. Thus, viral load in the CNS does not determine outcome to infection, but the stage of development of the animal has a significant impact on disease outcome [32]. This suggests that an immune mechanism may modulate disease outcome and the results suggest that JUNV is lymphotropic in the murine model. Mice that have been thymectomized at two to 24 hours after birth have 100% survival after JUNV challenge and have 10^7^ TCID50 virus in the brain [33]. Treatment with *Corynebacterium parvum* given simultaneously with Junín virus in mice increased survival time and the number of survivors [34]. *C. parvum* treatment could result in activation of macrophages, adjuncvanticity, depression of T cell function or increase in production of IFN. Interestingly, passive immunity conferred to suckling mice by natural (mainly through mother’s milk) or artificial means confer resistant to Junín virus infection [35].

JUNV studies in mice has shown T lymphocytes are essential for inducing neurological signs, lethality, antibody production, and the elimination of the virus. Fatal encephalitis is immunopathological and is not due to the direct action of the virus [36]. This is further illustrated when splenocytes from persistently infected immunocompetent mice were transferred into athymic mice and produced no signs of disease [37]. However, athymic mice infused with splenocytes from immunocompetent mice early in infection (6 to 30 days) experienced significant mortality [37]. Not only do immune T lymphocytes fail to effectively mediate Junín virus clearance in mice, they also appeared to mediate pathogenesis. T-cell mediated immunopathogenesis is hypothesized to occur in mice but not in other species, including humans. In humans there is an increase in antibody titers when viremia decreases and neutralizing antibodies confer protection. However, the same does not hold true with mice. Susceptible mouse strains infected i.c., the only route of inoculation that will produce symptoms in mice, induced a DTH reaction: in contrast, adult resistant BALB/c mice induced contrasuppressor cells (CS) to suppress DTH [38]. From the studies in mice we can see how T-cells may act in a pathogenic or protective manner in mice. However, these findings may not translate well to human disease. 

*In vitro* studies with pseduotyped virus suggested that tranferrin receptor 1 was most likely not the function receptor for mice [12]. However, mice can be infected with JUNV and even succumb to infection under immune modifications. A study showing Candid#1 infection in mouse cells was transferrin receptor 1 independent suggests an alternative receptor used for virus entry [33]. Perhaps an alternative receptor is used for the infection of mouse cells.

### 5.2. JUNV Infection in Guinea Pigs

In contrast, guinea pigs, an animal model more relevant to AHF, immunosuppressive drug treatments fail to protect animals from lethal infection with a virulent strain of JUNV; similar treatments even lead to fatal disease in guinea pigs infected with an attenuated strain of JUNV [39]. The distinct mechanisms underlying pathogenesis of JUNV infection of different animal models also highlight the importance of using a relevant model. At this time it is unknown if guinea pig transferrin receptor 1 can function as the cellular receptor for JUNV. Although, the cell tropism and clinical symptoms of JUNV infection in guinea pigs is similar closely mimics the human disease AHF without the complications of T-cell mediated pathogenesis as seen in mice [40,41,42]. Further research must be performed to elucidate the importance of transferrin receptor 1 in the guinea pig model. There is a linear dose-response for guinea pigs and mortality is based upon the dose administered i.p. to the animal [2]. However, strain dependent differences of JUNV show a range of virulence in guinea pigs from a fatal hemorrhagic syndrome to a neurological disease to a mild clinical disease [43]. Virus can be detected in the spleen and lymph nodes of guinea pigs independent of virulent virus strain at four-to-six days post infection (p.i.) [43]. However, the vaccine strain did not have detectable virus in any of the organs analyzed. The Kenyon study also revealed increasing virus titers in visceral organs in virulent strains of JUNV while other strains were neurotropic later in experimental infection and the animals exhibited neurological signs characterized by hind leg paralysis. Romero strain is virulent in inbred (Strain 13) and outbred guinea pigs (Hartley) but a passaged XJ strain is attenuated [44]. The XJ strain passaged several times in guinea pigs shows a neurotropism when administered intramuscular (i.m.) [45]. Guinea pigs infected with an attenuated strain of JUNV, XJCl3, had a 16% mortality rate with evidence of pancreatitis and meningoencephalitis [46].

### 5.3. JUNV Infection in Natural Host

The *Calomys* shed JUNV in their urine, saliva and feces and the animals develop a lifelong persistent infection. Although *C. musculinus* is the primary reservoir of JUNV, the virus is also occasionally isolated from *C. laucha*, *Akodon azarae*, and *Oryzomys flavescens *[47]. *C. musculinus* inoculated intranasally with JUNV at birth remain chronically infected for life and experience increased mortality, lower weight gain, and lower reproductive efficiency compared to uninfected animals [48]. Newborn *C. musculinus *inoculated with XJCl_3_ intraperitoneal (i.p.) animals experienced an acute disease with ataxia, tremors, and paralysis with 60% of animals succumbing to the disease and 30% resulting in chronic infection [49]. 

Horizontal transmission can occur and is thought to be the main form of dissemination [50]. *C. musculinus *populations have colonized fairly recently as shown by the high level of genetic subdivision within geographical region [51]. The expanding geographical distribution of JUNV could be due to the colonization of *C. musculinus*, the virus being able to infect other rodent species, the conversion of the pampas to farmland, or an explosion in the *C. musculinus* population due to pesticide use to control natural plants. Regardless of the reason, the expanding geographical distribution of Junín virus put more people at risk to contract the virus.

### 5.4. Junín Infection in Non-human primates

JUNV has been shown to produce a fatal disease in non-human primates that imitates natural infection in humans. The *Callithrix jacchus* (marmoset) has a completely lethal JUNV infection with primates succumbing to disease after the third week of infection with hemorrhagic and neurologic manifestations [52]. Treatment of non-human primates with immune serum significantly reduces mortality but produces a late neurologic syndrome similar to humans. There is a synergistic effect when Ribavirin is combined with immune plasma treatment in Junín virus infected *C. jacchus *[53]. Cross-protection of Tacaribe (TACV) infected *C. jacchus *was shown to confer protection to Junín virus when challenged 60 days after initial TACV infection [54]. The cross-protection of TACV infection was demonstrated through different routes of inoculations as well as long-term protection against virulent JUNV infection [55,56]. The safety, immunogenicity, and efficacy of Candid#1 was demonstrated in rhesus monkeys [57,58]. Non-human primates infected with arenaviruses showed detectable infectious virus in almost all visceral tissues, but the infection was accompanied by minimal histological damage [59].

## 6. Role of Adaptive Immune Response

JUNV infection generally causes immunosuppression in humans and animals. There is a decrease in the number of T and B lymphocytes, lower ratio of CD4 to CD8 T cells and diminished response to mitogens reported in acute AHF patients [60]. All subsets of lymphocytes return to normal levels in early AHF convalescence. Peripheral blood mononuclear cells (PBMCs) and polymorphonuclear cells perform normal antibody-dependent cell-mediated cytotoxicity (ADCC) [61], suggesting macrophages could be involved in virus clearance. Consistently, lymphoid necrosis in the spleen and impaired immunological competence were found in pathogenic JUNV infected guinea pigs [29,39].

Humoral immunity plays an important role in control of AHF. Infection of guinea pigs with attenuated strains induces neutralizing antibodies and protects animals from lethal challenge with pathogenic JUNV [29,62]. In immunosuppressed animals infected with attenuated JUNV and non-immunosuppressed animals infected with virulent virus succumb if due to failure to develop neutralizing antibodies and antibody dependent virus-specific cytotoxic spleen cell activity [39]. The critical role of antibodies in protecting animals from lethal infection is also supported by studies with non-human primates, demonstrated by the efficacy of immune serum in infected animals [53,63]. In humans, immune plasma from convalescence AHF patients is an effective treatment of AHF patients [64].

## 7. Vascular dysfunction

Hemorrhage manifestations are generally believed to be associated with thrombocytopenia, alteration in hemostasis and vascular dysfunction [65]. The exact role of the vascular endothelium in AHF is still largely unknown and may be distinct from some other VHFs. Ebola virus efficiently infects human endothelia cells and directly cause extensive cell damage [66], which could at least partially explain the increase in endothelium permeability and bleeding observed in patients and animal models. In contrast, JUNV infects human endothelium cells efficiently in cultured human umbilical vein endothelial cells (HUVECs) without causing apparent cytopathic effect [67,68], consistent with lack of specific vascular lesions in AHF fatal cases [69]. These observations suggest that JUNV infection of endothelial cells may not directly lead to disruption of blood and tissue barrier. JUNV infection induces endothelial cell dysfunction, including elevated expression of cell adhesion molecules ICAM-1 and VCAM-1, decreased prothrombotic von Willebrand factor, release of prostacyclin (PGI_2_) and decay accelerating factor (DAF) as well as upregulation of vasoactive mediator nitric oxide (NO) [68]. Importantly, both PGI_2_ and NO are endothelial derived anti-aggregatory factors regulating platelet activity. Alteration of endothelial cell function is more prominent in cells infected with virulent JUNV than that with non-virulent JUNV, suggesting a potential link between viral pathogenicity and the viral triggered alteration in endothelium function [68]. These results suggest a possible role of JUNV infection of endothelial cells in pathogenesis, which might be mediated by inducing release of cell mediators to perturb hemostasis and vascular integrity. Future studies with cell culture system and animal models are warranted to gain more evidence to characterize the role of endothelium dysfunction in AHF. 

## 8. Role of Glycoprotein in pathogenesis:

The JUNV envelope glycoprotein (GP) is a viral protein mediating receptor recognition and subsequent virus entry, which are mainly the functions of GP-1 and GP-2, respectively. The interplay of GP with host receptor determines virus host range, tissue tropism and consequently virus pathogenicity. JUNV, along with other New Word hemorrhagic fever arenaviruses, utilize transferrin receptor 1 (TfR1) to infect cells [70]. Expression of human TfR1, but not the TfR2, renders refractory hamster cell lines susceptible to infection by JUNV GP protein pseudotyped retrovirus. When TfR1 orthologs from different species are tested for their ability to support virus entry using pseudotyped retrovirus, TfR1 derived from *C. musculinus*, the natural rodent host of JUNV, most efficiently support JUNV GP mediated virus entry [71]. JUNV also uses human and domestic cat TfR1 orthologs efficiently, while TfR1 orthologs from house mouse and rat do not support virus entry. The latter discovery provides a reasonable explanation for the relative resistance of mice and rats to JUNV infection. The virus could be entering mouse and rat cells through a weak usage of TfR1 or through an alternative receptor to gain entry. Further detailed analysis has revealed that a region located on the apical domain of human TfR1 determines the efficiency of JUNV entry. Mutation in the counterpart region of mouse TfR1 with human TfR1 sequence converts mouse TfR1 an efficient receptor for JUNV entry [72]. These studies, the majority of the conclusions based on pseudotyped retrovirus model, are of great importance in the field and have clearly established the critical role of species-specific receptor usage in determination of JUNV host range, cellular tropism and pathogenicity. 

On the other hand, there are still many questions remaining regarding the role of interaction between GP and cellular receptor in viral pathogenesis. JUNV is known to infect murine derived cells and mice, which indicates that GP mediated virus entry is more complicated in JUNV infection than in pseudotype retrovirus infection. JUNV virus could infect murine cells via the mouse TfR1 or another yet unknown receptor, albeit with lower efficiency. Indeed, Candid#1 can infect and be propagated in different murine cell lines through a TfR1-independent mechanism [73]. In addition, since GPs from various strains of JUNV differs in their sequences, the effect of sequence diversity on receptor usage should be investigated. For example, the vaccine strain of JUNV, Candid#1 virus, was generated by inoculation of mouse brain for more than 40 passages. During this process, mutation in GP protein accumulated, accompanied by progressive attenuation for humans, guinea pigs and eventually mice. However it is unclear whether GPs from different strains of JUNV might exhibit, if any, variation in receptor recognition. Candid#1 GP has an increased dependence on human TfR1 [74], suggesting the potential contribution of GP sequence divergence to JUNV attenuation.

In addition to the effect on receptor recognition, mutation in GP sequence might also affect virus entry as a result of alteration in GP fusion activity. A F427I substitution in the transmembrane region of GP2 of the JUNV has been identified as a major determinant of attenuation in mice [75]. In a reporter minigenome system, the F427I mutation reduces virus infectivity by affecting GP fusion activity, likely due to the proposed destabilization of GP metastable conformation [74]. This mutation emerged at the late stage of continuous passage of pathogenic JUNV in mouse brain and cultured cells and has been shown to contribute to virus attenuation in mice. Further studies introducing the same mutation in a pathogenic strain could lead to virus attenuation in more relevant an animal model that more closely mimics the pathogenesis observed in humans, such as guinea pigs. This study will facilitate identification of genetic markers for JUNV attenuation. At this time it is unknown the affect F427I will have on human cells 

## 9. Target cells and cell entry

Studies of fatal cases and animal models of AHF have identified monocytes, macrophages and dendritic cells as JUNV primary cell targets [17,76,77]. Thus, an infectious center assay rendered positive results for PBMCs but not for lymphocytes when tested from 2 to 12 days symptomatic AHF patients. Similarly, co-cultivation of PBMCs with Vero monolayers allowed JUNV detection in 96% cases of acute AHF [76]. Moreover, JUNV production was observed in *ex vivo* cultures of PBMCs only when adherent mononuclear cells were present [76,77]. Additionally, the presence of JUNV antigen and virus budding through immunostaining and electron miscopy was detected in reticular/phagocytic but not lymphocytic cells of spleen and lymph nodes from 7 fatal cases of AHF [17]. 

At 5 days p.i., 2 days earlier then viremia was detected, virus production, shown through plaque assay, was observed in bone marrow of JUNV XJ-infected guinea pigs. Starting at 7 days p.i. the viral load in lymphoid tissues (bone marrow, lymph nodes and spleen) was higher of that in blood. At the same time, JUNV antigen was observed by immunostaining in reticular monocytes and megakaryocytes but not in lymphoid cells of spleen, lymph nodes and bone marrow of guinea pigs infected with the virulent strain XJ of JUNV. At 5-7 days p.i. virus like particles were observed by electron microscopy in the rough endoplasmic reticulum (RER) of bone marrow reticular cells [40].

Pathogenic JUNV strain XJ (uniformly lethal in guinea pigs) was detected by immunostaining or isolated through intracerebral inoculation into newborn Swiss mice from macrophages and dendritic cells purified from spleens of infected animals starting as early as 4 days p.i. until death at day 11-14 p.i. In contrast, partially attenuated XJCl3 virus (16% lethality in guinea pigs) was detected in the dendritic cells purified from the infected animals starting at 7 days p.i. [78,79]. Similar to XJCl3 observations were made for non-pathogenic Tacaribe virus. JUNV XJ or XJCl3 production was not detected in *ex vivo*-infected dendritic cells purified from spleens of Swiss, BALB/c, and C57BL/6J mice in 24 h p.i.; however 30% of cells were viral antigen positive by immunohistochemical staining. Comparable IFN activity was detected in the supernatant of these cells in response to both viruses [79]. In one another study, XJCl3 or Candid#1 production was detected in both adherent and non-adherent populations of PBMCs purified from guinea pig or *C.musculinus* 2–26 or 5–15 days p.i. respectively [80,81]. In addition, replication of JUNV has been observed in human monocytes and macrophages [82], as well as rat [83] and *C. musculinus* [84] macrophages cultures *ex vivo*. 

Silica treatment studies confirm an important role of macrophages in JUNV pathogenesis. Thus, i.p. infection of JUNV pathogenic strain XJ in newborn rats is almost uniformly lethal with neurological involvement, while attenuated XJCl13 infection has a mortality rate of 15%. Upon macrophage depletion with silica brain spread and the mortality rate of XJ-infected two day old rats drastically decreased. Surprisingly, treatment did not affect the mortality of XJCl13-challenged animals. Additionally, XJ infection became lethal (36% mortality rate) in normally virus-resistant adult rats upon pretreatment with silica [85]. Moreover, depletion of mononuclear phagocytes with silica treatment drastically reduced the Candid#1 neuroinvasion in *C. musculinus* [80].

## 10. Innate Immune Response

High serum levels of endogenous IFN-α (16,000-64,000 IU/mL) have been detected in patients with AHF in the acute stage of disease which correlates with severity of disease [86]. Correlation of IFN-α activity with fever, chills, backache and reverse correlation with days of disease evolution has been observed for AHF patients [87]. High endogenous levels of IFN-α in AHF also correlate with low platelet count and platelet abnormality [88]. Recent *in vitro* study of human CD34^+^ cell treated with thrombopoetin demonstrated decreased platelet formation, release and function upon JUNV infection via bystander effect mediated by type I interferon signaling; while apoptosis, proliferation, clonogenic ability and maturation of this cells were not affected [89]. Induction of IFN-α correlated with JUNV infection has also been documented in animal models of AHF [90,91]. 

Elevated levels of IL-6, IL-8, IL-10 and TNF-α were detected in AHF patients in severe, moderate and mild cases related to severity of the disease [92]. Monocytes and macrophages are unlikely souse of these inflammatory cytokines in human infections. Thus, no elevated production of IFN-α, IFN-β, IL-6, IL-10, IL-12, or TNF-α was detected in cultures of PBMCs purified human monocytes and macrophages infected with JUNV pathogenic Romero strain. However, IL-6, IL-10, and TNF-α levels were increased in response to non-pathogenic arenavirus closely related to JUNV, TACV [82]. 

Absence of pro-inflammatory response in JUNV infected monocytes and macrophages could be explained by the data generated upon overexpression of JUNV proteins in the cell-based gene reporter assays [93,94,95,96,97,98]. Thus, interference with IFN-I induction in response to RIG-I like helicase (RLH) pathway stimulation has been documented for JUNV NP and Z proteins in cell-based gene reporter assays. For instance, an overexpression of JUNV NP has been shown to blocks interferon regulatory factor (IRF)-3 activation and nuclear translocation resulting in transcription inhibition of IRF-3 or IFN-β responsive genes in response to Sendai virus [96]. Inhibition of IFN-β transcription and IRF-3/NF-κβ activation in response to 5' triphosphated RNA were detected in presence of JUNV Z protein associated with Z protein interference with mitochondrial antiviral-signaling protein (MAVS)/RIG-I interaction. Additionally, binding of Z protein of JUNV to RIG-I was demonstrated through co-immunoprecipitation and confocal microscopy [98]. Moreover, recently, co-immunoprecipitation of (IKK)-related kinase IKKε and JUNV NP has been demonstrated leading to inhibition of NF-κβ promoter activity and p65 nuclear translocation. In the same experiments NP of LCMV has been shown to bind and inhibit the kinase domain of IKKε resulting into blockage of both IRF-3 and NF-κβ activation. [93,94]. 

JUNV infection of parenchymal cells in the acute stage of AHF might be the source of high levels of IFN-α. Human lung carcinoma cells (A549) infected with either virulent (Romero) or vaccine (Candid#1) strains of JUNV induced IFN-I production, interferon stimulated gene (ISG) expression and Signal Transducer and Activator of Transcription (STAT)1 phosphorylation. Additionally, RIG-I was demonstrated as a primary trigger of IFN-I signaling in response to JUNV infection in these cells. Thus, siRNA-mediated downregulation of RIG-I or IRF-3 production obliterated STAT1 phosphorylation in Candid#1 infected A549 cells [99]. The apparent discrepancy of the live virus data with the aforementioned cell-based gene reporter assays may relate to the fact that plasmid-driven overexpression of JUNV NP and Z proteins preceded RLH stimulation. Whereas at early times during JUNV infection NP and Z protein levels may not be sufficient to counteract activation of the RLH pathway leading to induction of IFN-I [93].

In contrast to JUNV infection of human reticular cells, both vaccine (Candid#1) and VLP pseudotyped with GPs of pathogenic (Parodi) strains of JUNV induce production of IFN-β and TNF-α from mouse macrophage cell lines and primary macrophages. This induction of pro-inflammatory cytokines was viral replication- and RLH-pathway-independent but TLR2 dependent [73]. The results of this study suggest that innate immune response of a mouse to JUNV may be species specific. 

Surprisingly, JUNV production in Vero cells was rather resistant to the antiviral effects of pretreatment with IFN-I and II [99]. Similar results were observed by others [100,101]. Adult mice are resistant to JUNV-induced disease when challenged peripherally. However, mice (strain 129 and C57BL/6 hybrid) deficient for alpha/beta and gamma interferon receptor developed severe disease and disseminated infection following i.p. inoculation with JUNV Romero. Additionally, the virus production was comparable in mouse embryonic fibroblasts (MEFs) derived from wild type and deficient animals [102]. These data suggest a significant role of IFN-I for development of innate or adaptive immune response to JUNV. 

## 11. Live-attenuated vaccines

A heterologous New World arenavirus, TACV, has been evaluated for the ability to induce cross-protection against JUNV in guinea pigs and NHPs. Guinea pigs immunized with a single dose of TACV were fully protected against JUNV [103]. Further studies in guinea pigs showed complete clearance of the virus by day 30 after TACV infection. Anti-TACV neutralizing antibodies capable of cross-reacting with JUNV were detected up to two years and full protection against JUNV challenge was achieved at up to 18 months after initial immunization with TACV [104]. Marmosets inoculated with TACV failed to induce a detectable disease as evident by normal blood parameters such as erythrocyte, leukocyte, reticulocyte, and platelet counts) as well as hematocrit or hemoglobin values and no detectable viremia. However, the marmosets developed neutralizing antibodies three weeks postinfection. The TACV-immunized marmosets were challenged with the pathogenic XJ strain of JUNV, which resulted in the complete protection of the experimental animals from the lethal challenge compared to the control animals that developed AHF [54]. These studies indicate that TACV could be used as a platform for the development of vaccines against JUNV and other New World arenaviruses. 

Several live-attenuated JUNV strains have been derived from the prototype XJ strain of JUNV isolated from humans by Parodi *et al* [105] by passaging in cultured cells, mouse brains and guinea pigs. XJCl3 strain was derived by plaquing XJ strain in MA-111 (rabbit kidney) cells. XJCl3 was significantly attenuated in guinea pigs and mice compared to the parental XJ strain and elicited high titers of neutralizing antibodies in inoculated animals [106]. A vaccine formulation based on XJCl3 strain was administered to 636 human volunteers, most of whom developed a subclinical infection or mild symptoms. The immunization induced high titers of neutralizing antibodies that were detectable for up to nine years post-immunization in 90% of tested vaccine recipient (153 out of 165) [107]; persistence of antibodies and clinical evaluation in volunteers 7 to 9 years following the vaccination against AHF. However, since the seed virus vaccine stock of XJCl3 strain was established in suckling mouse brain and cloned in a heteroploid cell line the clinical trials were interrupted. 

Another live-attenuated JUNV strain XJ0 was derived from a common parental virus with XJCl3. Immunization of guinea pigs with XJ0 strain induced protective immunity against pathogenic JUNV challenge starting as early as three days p.i. and at 30 days protected 100% of experimental animals [108]. Although, these result demonstrated the potential of XJ0 strain for development of a live-attenuated vaccine the studies were discontinued when persistent JUNV infection was detected in lymphohemopoietic organs of inoculated guinea pigs (reviewed in [109]). 

Candid #1 vaccine strain of JUNV was developed as a collaborative effort by the US Army Medical Research Institute of Infectious Diseases (USAMRIID) and the Argentine Ministry of Health and Social Action. Candid #1 has a defined passage history (Fig. X), which originated from the prototype XJ strain of JUNV through two initial passages in the guinea pig followed by 44 passages in the mouse brain. The brain homogenate from the last mouse brain passage was used to infect FRhL-2 cells (certified fetal rhesus lung diploid cell line), in which Candid #1 was passaged twelve times followed by cloning through two limiting dilution steps. The master and secondary seeds were obtained after a single amplification round in FRhL-2 cells. The vaccine stock of Candid #1 was produced by a single amplification of the secondary seed resulting in the total of 19 passages in FRhL-2 cells (reviewed in [110]). The high protective efficacy and the lack of neurovirulence of Candid #1 was demonstrated in guinea pigs and NHPs [58,111]. The field tests aimed at the evaluation of the immunogenicity and efficacy of Candid #1 in humans involving 6,500 male agricultural workers in AHF-endemic region of Argentina demonstrated high protective efficacy of Candid #1 (≥ 84%). In addition no serious adverse effects were detected in association with vaccination [112]. Currently Candid #1 is the only vaccine that is fully approved for use in Argentina for the prevention of AHF and has been used to vaccinate more than 200,000 people at risk. 

**Figure 2:**
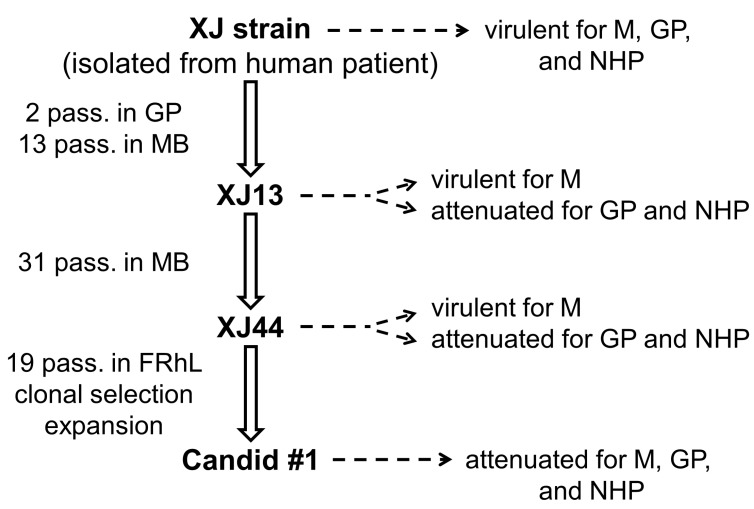
Development of Candid #1 vaccine strain of JUNV (adapted from Albarino *et al*., 2011). M = mouse, MB = mouse brain, GP = guinea pig, NHP = non-human primate, FRhL = fetal rhesus monkey lung cells.

## 12. Other vaccine preparations

The glycoproteins of JUNV were identified as a major immunogen [113]. Various sub-viral components were extracted from samples containing purified virus treated with Triton X-100 nonionic detergent. However, only glycoprotein containing soluble fraction was immunogenic in laboratory animals and induced formation of neutralizing antibodies that protected them against subsequent challenge. Immunogenicity and protective properties of formalin-inactivated JUNV vaccine preparations have also been evaluated. The XJCl3 strain of JUNV grown in Vero cells was subjected to formalin inactivation at 0°C followed by concentration with polyethylene glycol (PEG). Immunization of guinea pigs with the prepared antigens resulted in the development of high neutralizing titers; however, no protection was gained against the following challenge with the with the highly pathogenic XJ strain [114]. These results suggested that alternative methods of antigen preparation and/or delivery may be required to elicit protective immunity. This notion was later supported by a study where an alphavirus-based replicon system based on a human (United States Food and Drug Administration Investigational New Drug status) vaccine TC83 was used to express the glycoproteins of Candid #1 strain of JUNV. A single dose of the live-attenuated alphavirus-based vaccine expressing only GPC was immunogenic in guinea pigs and provided partial protection, while a double dose of the same vaccine provided complete protection against Romero strain of JUNV that is highly virulent in guinea pigs [115]. Therefore, the immune response against GPC alone is sufficient to prevent lethal disease caused by JUNV infection. 

## 13. Therapeutics

Treatment with immune plasma from previously infected patients is the standard treatment available against AHF. Published case series have shown the reduction of mortality to 1-2% in patients that were administered immune plasma within eight days of the onset of clinical illness [116]. Eight to 10% of patients treated with immune plasma develop late neurologic syndrome (LNS), which may include ataxia, nystagmus, cerebellar tremors, fever, cerebellar, cranial nerve palsies and gait laterization (changes in walking patterns). Interestingly, there are no known cases of LNS to date in patients with AHF who recovered without treatment [116]. The exact mechanism and cause of LNS remains unknown.

The ability of purified IgG to protect from adverse outcome *in vitro* and *in vivo* was studied to determine the exact protection mechanism of immune serum. Purified immune plasma fractions IgG_1,2,4_, IgG_1,2,3,4_ and F(ab’)_2_ could neutralize Junín virus *in vitro* but the F(ab’)_2_ fraction failed to protect guinea pigs from death when the animals were administered 6,000 therapeutic units (TU). In contrast, an equivalent dose of either the IgG_1,2,4_ or IgG1_,2,3,4_ fraction was able to protect guinea pigs from lethal challenge [117]. The failure of purified F(ab’)_2_ to protect animals despite its ability to neutralize virus *in vitro* shows that virus neutralization is not sufficient to protect animals. These data suggest that clearance of infected cells is a very important mechanism in protection.

There are several reasons to develop alternative therapies for AHF including the lack of efficacy of immune plasma beyond the first eight days post onset of symptoms, the risk of transfusion-borne diseases, the possibility of developing LNS, and the practical difficulties of finding immune serum donors [64]. The only approved treatment for arenaviruses in the US is Ribavirin, a nucleoside analog that has been shown effective as an antiviral against different viral diseases [118]. NHP testing using rhesus macaques showed that clinical disease can be prevented if Ribavirin is given at the time of challenge but if treatment is begun at six days p.i. only results in a delay of time of death. Ribavirin has also shown to be beneficial for the treatment of Bolivian hemorrhagic fever (BHF) in NHPs [119]. Ribavirin has been used to treat several patients including a laboratory exposure of arenaviruses [120,121]. It has also been shown to be effective at treatment of AHF and BHF if given to patients early in the course of the disease [122,123]. Studies in the guinea pig model have shown that Ribavirin can increase the mean time until death and delay viral replication when administered subcutaneously. Ultimately, however, animals succumbed to the disease despite antiviral therapy, regardless of the route of infection [124]. Recent studies have shown that a high dose of Ribavirin over a long period of time is able to statistically increase survival of JUNV-infected guinea pigs [125]. 
